# Impact of fluid overload on blood pressure variability in patients on peritoneal dialysis

**DOI:** 10.1080/0886022X.2022.2148535

**Published:** 2022-11-22

**Authors:** Yuanmeng Jin, Xiaomin Huang, Chunyan Zhang, Jingyuan Xie, Hong Ren

**Affiliations:** a Department of Nephrology, Ruijin Hospital, Shanghai Jiao Tong University, School of Medicine, Shanghai, P.R. China; b Institute of Nephrology, Shanghai Jiao Tong University School of Medicine, Shanghai, P.R. China

**Keywords:** Blood pressure variability, body composition monitor, peritoneal dialysis, volume, overload values

## Abstract

Fluid overload is a common complication in patients who are on continuous ambulatory peritoneal dialysis (CAPD). Blood pressure is the traditional indicator of fluid status in these patients. However, it has poor sensitivity. Blood pressure variability (BPV) can detect fluctuations in blood pressure sooner and more accurately and be useful for the assessment of fluid volume in hemodialysis patient. However, there are limited relevant studies involving patients on CAPD. This retrospective study included 175 patients on CAPD who underwent regular assessment of the peritoneal dialysis adequacy at 2–3-month intervals at our center from January 2018 to December 2020. The overhydration (OH) value was measured using a body composition monitor. Blood pressure measurements obtained in each visit over one-year follow-up were used to determine each patient’s BPV under peritoneal dialysis. The patients were divided into the normal volume (OH ≤2 L), mild volume overload (OH 2–4 L), and severe volume overload groups (OH ≥4 L). There was no significant between-group difference in the mean blood pressure (*p* > 0.05). However, the systolic and diastolic BPV values were significantly higher in the severe volume overload group than in the other groups (*p* < 0.05). Further correlation and regression analysis showed volume overload severity and BPV existed positive association, and BPV was also significantly associated with PD volume related indexes, including diabetes mellitus, blood parathyroid hormone levels, Kt/V and subjective global assessment scores in the volume overloaded group (*p* < 0.05). All these results suggested BPV may be a useful indicator for fluid status in PD patients.

## Introduction

Continuous ambulatory peritoneal dialysis (CAPD) is widely used as a renal replacement therapy in end-stage renal disease. Volume overload is a common complication of CAPD with an incidence of more than 60%, which is much higher than that in patients on hemodialysis. Previous studies have shown that volume overload is an independent risk factor for cardiovascular disease and mortality in patients with CAPD. However, it is difficult to detect and evaluate retention of water and sodium in patients on peritoneal dialysis (PD) [1–3].

Hypertension is highly prevalent in patients on PD, and volume overload is the main cause of uncontrolled hypertension in these patients. Therefore, the International Society for Peritoneal Dialysis guidelines recommend volume control as the first-line treatment for hypertension in this patient population. In clinical practice, blood pressure (BP) levels are routinely used to evaluate volume status in patients on CAPD. Other indicators, including edema, urine volume, ultrafiltration volume, and body weight changes, are ever used to guide early interventions but showed poor specificity and sensitivity [4–6].

Blood pressure variability (BVP) reflects fluctuations in BP within a specific period of time. Previous studies have shown that BPV is a more sensitive indicator of volume overload than absolute BP in patients on hemodialysis and a better predictor of target organ damage and mortality [7,8]. However, there is less relevant data for patients on PD.

Body composition monitoring is an accurate and noninvasive way of measuring a patient’s intracellular fluid, extracellular fluid, and overall water content and calculates the overhydration (OH) value. The precision of this approach is close to that of the accepted isotope dilution method [9]. Previous studies have suggested that the upper limit of OH indicating stable fluid status is 2.0 L in Chinese patients on CAPD [10]. The aims of this study were to explore the relationship between OH values and long-term BPV in patients on PD and to determine the role of BPV in assessment of volume status in these patients.

## Materials and methods

### Study design

One hundred and seventy-five patients who underwent CAPD at Peritoneal Dialysis Center of Ruijin Hospital from January 2018 to December 2020 were retrospectively enrolled. All patients were over the age of 18 years and had received regular PD for >6 months. They accepted regular assessment of the adequacy of PD at 2–3-month intervals, as well as physical examinations and clinical assessments (including blood pressure measurement, observation of edema, urine volume, weight, dyspnea, et al.) to estimate hydration status. The exclusion criteria were as follows: (1) placement of coronary stents or a pacemaker; (2) a metal implant, such as an artificial joint; (3) contralateral or bilateral amputation; (4) severe heart failure (New York Heart Association Class IV); and (5) severe acute infectious disease or malignant tumor. All patients were treated with a lactate-based dialysate (Baxter Healthcare, Deerfield, IL, USA) with an exchange volume of 6–8 L/day [11,12]. The OH value was measured using a body composition monitor (Fresenius Medical Care, Wendel, Germany). Long-term BPV was calculated using the BP data obtained during routine clinical visits[13]. The research was conducted following the World Medical Association Declaration of Helsinki. The study protocol was approved by our institutional ethics committee [ETHICS No: (2020) Linlun no. 43th]. Subjects have given their oral and written informed consent.

### Collection of general data

All patients had complete baseline demographic information, medical history, and regular follow-up data, including measurements for weight, height, systolic and diastolic BP, urine volume, ultrafiltration volume, body mass index and laboratory test results. Blood, urine, and PD fluid samples were obtained using consistent techniques. All laboratory values were measured using automated and standardized methods. Appropriate clinical treatment was provided in all cases.

### Assessment of fluid status and patient groups

At the last visit of the year, BCM measurements were performed in the morning before the infusion of peritoneal dialysis fluid. The patients sit relaxed in the dialysis chair for at least 5 min. Bioimpedance spectroscopy was performed using the body composition monitor (BCM, Fresenius Medical Care, Wendel, Germany), strictly according to the manufacturer’s recommendations. Electrodes were attached to one hand and one foot at the ipsilateral side.The BCM uses alternating electric currents at 50 different frequencies (5-1,000 kHz) to measure fluid status.The OH value is calculated on the basis of the physiologic tissue model that comprises the individual’s normal ECW, normohydrated lean and adipose tissue. The OH value can be calculated from the difference between the normal expected ECW and the measured ECW [12]. Patients were divided into a normal volume group (OH ≤ 2L), a mild volume overload group (2 L < OH < 4L), and a severe volume overload group (OH ≥ 4L).

### Measurement of BP and BPV

At each visit in our PD center, the blood pressures were routinely measured by renal clinicians or nurse specialists before the start of daily peritoneal dialysis. The mean values of the systolic and diastolic blood pressure were recorded, and the standard deviations (SD) were calculated. BPV was defined as the coefficient of variation (standard deviation/mean BP*100), which had been validated in previous studies of dialysis population.

### Statistical analysis

The data for categorical variables are expressed as numerical and percentage values. Data for continuous variables are expressed as the mean ± SD. Between-group comparisons were performed using the *t*-test for continuous variables and the chi-squared test for categorical variables. Linear regression analysis was conducted to determine the associations between OH values and BPV, including BPV of systolic blood pressure (SBP) and diastolic blood pressure (DBP). Data for the continuous variables were analyzed in univariable and multivariable models, and hazard ratios and 95% confidence intervals per SD are presented for each parameter. Multiple linear regression was used to identify determinants of BPV. All statistical analyses were performed using SPSS software (version 20.0; IBM Corp., Armonk, NY, USA). *P*-value of <0.05 was considered statistically significant.

## Results

### Baseline characteristics

**Figure 1.**
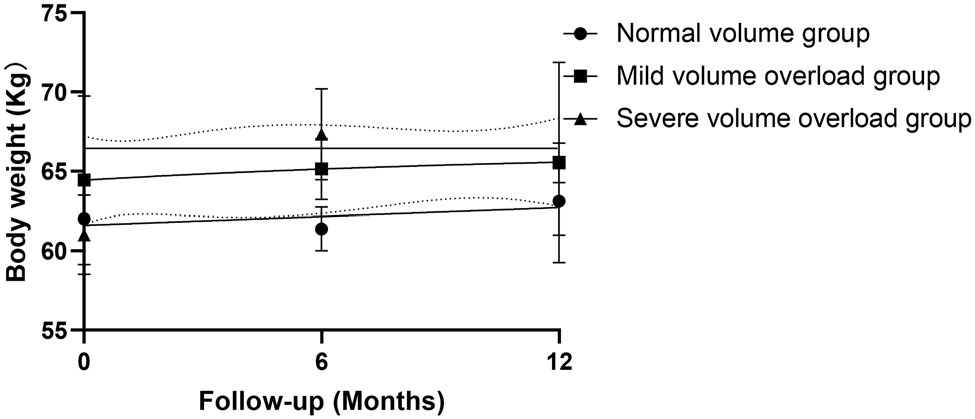
Body weight changes during 1-year follow-up.

One hundred and one (57.7%) of the 175 study participants were male (median age 52.6 years) and 74 (42.3%) were female (median age 53.6 years). There were 91 patients (52%) in the normal volume group, 51 (29%) in the mild volume overload group, and 33 (19%) in the severe volume overload group. The patient demographic characteristics were presented in Table 1. Body weight changes in one-year follow-up were presented in Figure 1.

**Table 1.** Clinical data at baseline in different volume groups (*N* = 175).

Parameters	Normal volume (O*H* ≤ 2L)	Mild overload (2L < O*H* < 4L)	Severe overload (O*H* ≥ 4L)	*p* Value
	*N* = 91	*N* = 51	*N* = 33	
Age (years)	53.7 ± 15.7	54.4 ± 13.4	52.6 ± 11.3	0.04
BMI per (kg/m^2^)	23.1 ± 9.9	22.8 ± 2.6	23.3 ± 3.2	0.15
Hemoglobin (g/L)	114.4 ± 14.7	108.9 ± 13.5	102.8 ± 16.9	<0.01*
Albumin (g/L)	36.1 ± 4.3	33.7 ± 3.7	34.7 ± 4.1	0.15
Triglycerides (mmol/L)	2.1 ± 1.2	1.6 ± 0.6	1.8 ± 1.2	0.06
Cholesterol (mmol/L)	4.9 ± 0.9	4.4 ± 1.1	4.9 ± 1.1	0.97
Uric acid (mmol/L)	375.9 ± 73.2	379.8 ± 59.8	384.6 ± 72.1	0.45
Calcium (mmol/L)	2.3 ± 0.2	2.4 ± 0.1	2.3 ± 0.2	0.42
Phosphorus (mmol/L)	1.4 ± 0.4	1.6 ± 0.5	1.7 ± 0.5	<0.01*
PTH (pg/mL)	253.3 ± 203.1	280.9 ± 271.1	326.2 ± 236.1	<0.01*
Sex (male)/n (%)	47 (51.6%)	30 (58.8%)	28 (84.8%)	<0.01*
Diabetes/%	6 (6.60%)	5 (9.80%)	7 (21.20%)	<0.01*
SGA scores/%	86.10%	71.80%	54.10%	0.03*

BMI: body mass index; PTH: parathyroid hormone; OH: overhydration; SGA: subjective global assessment. *P*-values refer to the overall difference across categories. *p < 0.05 was considered statistically significant.

### Changes in BP and BPV

**Figure 2.**
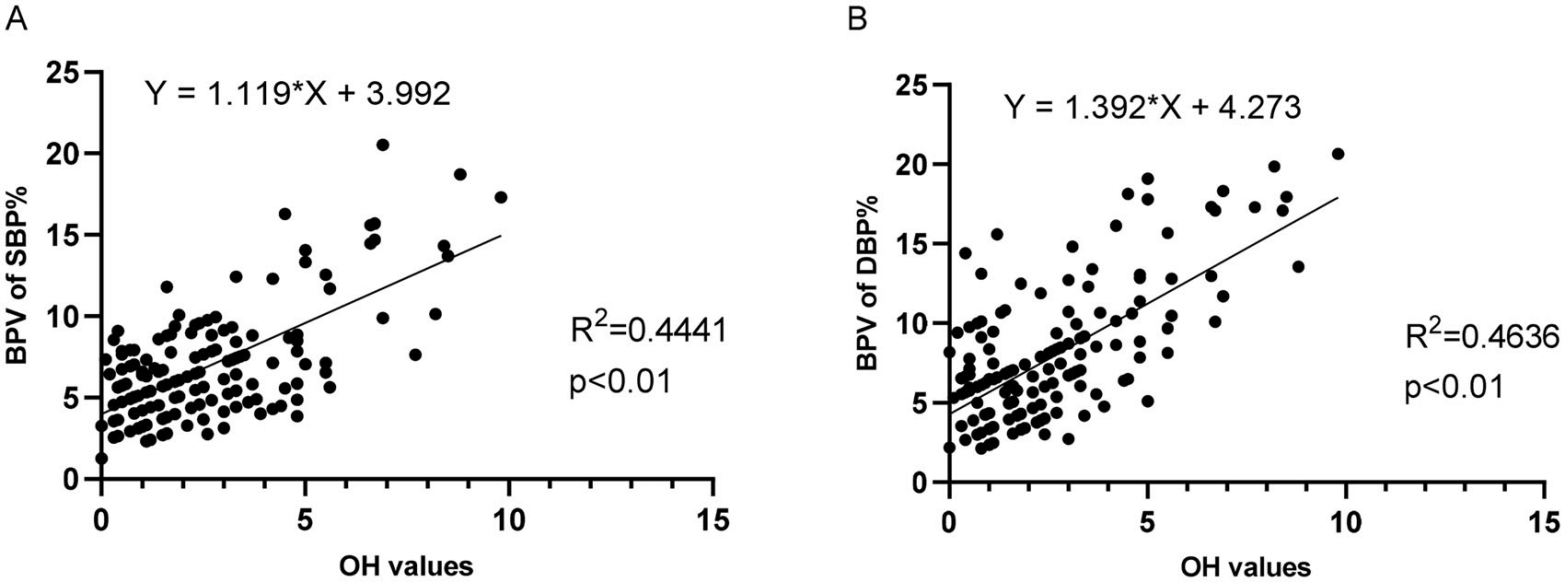
Linear regression for the association between OH values and BPV of SBP% (A) and DBP% (B).

The mean systolic and diastolic BP values were no significant between-group differences (*p* > 0.05). But respective systolic and diastolic BPV values in severe volume overload groups were significantly higher than other two groups. The results of the BP variability parameters were shown in Table 2. Linear regression supported that OH was associated with BPV and overall performance of the linear regression models were 0.44 for SBP and 0.46 for DBP (Figure 2).

**Table 2.** Comparation of blood pressure and variability in different groups.

Parameters	Normal volume	Mild overload	Severe overload	*p* Value
	(O*H* ≤ 2L)	(2L < O*H* < 4L)	(O*H* ≥ 4L)	
	*N* = 91	*N* = 51	*N* = 33	
**SBP (mmHg)**	137.8 ± 14.2	142.1 ± 7.5	151.8 ± 9.8	0.06
**DPB (mmHg)**	83.3 ± 16.3	83.4 ± 3.5	83.5 ± 7.1	0.08
**BPV of SBP%**	7.76 ± 2.93%	9.23 ± 3.33%	12.74 ± 6.34%*	<0.01*
**BPV of DBP%**	7.72 ± 3.16%	8.7 ± 5.43%	12.22 ± 6.12%*	<0.01*

SBP: systolic blood pressure; DPB: diastolic blood pressure; BPV: blood pressure variability; OH: overhydration. *P*-values refer to the overall difference across categories. *p < 0.05 vs. normal volume group (OH ≤ 2L).

### Clinical index scores according to volume group

Examination of the clinical data obtained during the study showed that the severe volume overload group contained significantly more patients with diabetes mellitus (21.2%) and men (84.8%). Serum levels of phosphorus and parathyroid hormone were significantly higher in this group than in the other groups (Table 1). The mean patient age, hemoglobin value, Kt/V, peritoneal creatinine clearance rate (CCr), subjective global assessment (SGA) score, urine output(L/day), total fluid removal(L/d) were significantly lower in the severe volume overload group (Tables 1 and 3). Additionally, the prescribed diuretics% and numbers of antihypertensive drugs were higher (Table 3). Other indexes, including body weight changes in 1-year follow-up, showed no significant difference among groups.

**Table 3.** Peritoneal dialysis related parameters and medications(*N* = 175).

	Normal volume (O*H* ≤ 2L)	Mild overload (2L < O*H* < 4L)	Severe overload (O*H* ≥ 4L)	*p* Value
	*N* = 91	*N* = 51	*N* = 33	
**Dialysis-related parameters**				
Fast plasma glucose(mmol/L)	4.9 ± 1.6	4.8 ± 1.9	5.2 ± 2.1	0.30
Glucose exposure(g/24h)	142.5 ± 25.2	150.8 ± 24.8	159.3 ± 19.2	0.25
Total Kt/V	2.48 ± 0.84	2.12 ± 0.43	2.0 ± 0.5	<0.01*
CCr (ml/min)	69.24 ± 24.92	56.30 ± 11.20	50.30 ± 17.40	<0.01*
Residual GFR (ml/min)	2.39 ± 0.74	2.10 ± 0.29	2.0 ± 0.14	<0.01*
Transport status				
(high average%)	24 (24.6%)	16 (31.4%)	15 (46.2%)	<0.01*
nPCR (g/kg/day)	0.98 ± 0.23	0.89 ± 0.21	0.84 ± 0.17	<0.01*
Fluid removal				
Urine output(L/day)	0.64 ± 0.4	0.25 ± 0.14	0.17 ± 0.13	<0.01*
Ultrafiltration(L/day)	0.57 ± 0.28	0.83 ± 0.31	0.82 ± 0.38	0.32
Total fluid removal				
(L/day)	1.21 ± 0.43	1.08 ± 0.31	0.99 ± 0.28	0.04*
**Drugs**				
Prescribed diuretics (%)	12 (13.2%)	9 (17.6%)	9(27.2%)	<0.01*
Numbers of antihypertensive drugs	1.9 ± 1.7	3.2 ± 1.1	3.7 ± 0.9	<0.05*

Kt/V: urea clearance index; Ccr: creatinine clearance; nPCR: normalized protein catabolic rate; OH: overhydration. *P*-values refer to the overall difference across categories. *p < 0.05 was considered statistically significant.

### Analysis of risk factors for BPV in three groups

In correlation analysis, clinical indexes including serum phosphorus, PTH, Kt/V, CCr, nPCR, SGA scores, diabetes status, transport status (high average%) correlated well with BPV in different volume groups (Table 4). We further performed multiple linear regression analysis to evaluate risk factors on the basis of correlation analysis. Four factors-diabetic status, Kt/V, serum PTH, SGA scores were independently associated with BPV. Higher BPV positively associated with diabetes status and serum PTH levels and negatively associated with the adequacy of dialysis (Kt/V) and SGA scores (Table 5).

**Table 4.** Parameters related to BPV in different volume groups.

Parameters	OR (95% CI)	*p* Value
**Diabetes/%**	0.249 (0.116–0.537)	<0.01*
**Phosphorus (mmol/L)**	0.323 (0.185–0.563)	<0.001*
**PTH (pg/mL)**	0.372 (0.214–0.647)	<0.001*
**Kt/V**	3.403 (1.608–7.199)	<0.01*
**CCr**	0.426 (0.238–0.760)	<0.05*
**nPCR**	3.776 (1.940–3.747)	<0.001*
**SGA scores**	0.155 (0.086–0.282)	<0.001*
**Transport status**		
**(high average%)**	0.198 (0.102–0.491)	<0.001*

PTH: parathyroid hormone; OR: odds ratio; Kt/V: urea clearance index; Ccr: creatinine clearance; nPCR: normalized protein catabolic rate; SGA: subjective global assessment; BPV: blood pressure variability; CI: confidence interval; OH, overhydration. *p < 0.05 was considered statistically significant.

**Table 5.** Multivariate linear regression of BPV associated parameters.

Parameters	β	*p* Value
**Diabetes status**	0.278	<0.01*
**Phosphorus (mmol/L)**	−0.070	0.83
**PTH (pg/mL)**	0.180	<0.05*
**Kt/V**	−0.720	<0.01*
**CCr(ml/min)**	0.299	0.26
**nPCR**	0.047	0.79
**SGA scores**	0.897	<0.01*
**Transport status** **(high average%)**	0.023	0.23

PTH: parathyroid hormone; β: standardized regression coefficient; Kt/V: urea clearance index; Ccr: creatinine clearance; nPCR: normalized protein catabolic rate; SGA: subjective global assessment; BPV: blood pressure variability; OH: overhydration. *p < 0.05 was considered statistically significant.

## Discussion

Part of PD patients are prone to over-hydrated status which usually progress slowly and not easily to be detected when compared to hemodialysis patients. These fluid overloads can lead to a range of complications, including increasing cardiovascular events, which seriously affect the prognosis of PD patients. Therefore, early detection of fluid overload is important. Brain-type natriuretic peptide has been validated to correlate well with fluid overload in dialysis patients [14], but other indicators of fluid status are still needed to be studied. In this study, we preliminarily validated long-term BPV was associated with fluid status in PD patients. Moreover, BPV was positively associated with diabetes status and blood PTH levels and negatively associated with the adequacy of dialysis (Kt/V) and SGA scores in the volume overload group.

Fluid overload is an important factor in patients on CAPD who have uncontrolled hypertension, pulmonary and peripheral edema, heart failure, or other cardiovascular complications. However, traditional BP monitoring cannot detect a potential increase in volume status over time. BPV has recently been confirmed as a more sensitive method for reflecting fluctuation in homeostasis of BP, even in patients with “normal” BP. Several studies, particularly in the hemodialysis population, indicate that BPV is an independent risk factor for cardiovascular events and mortality. However, less studies have been performed in PD patients [15–18].

BPV consists of short-term, medium-term, and long-term BPV (egvisit-to-visit BPV). Long-term monitoring of BPV requires a lengthy period of time, but could avoid interruption of environments and temperatures [14]. In clinical practice, BPV is known to reflect actual BP status more concisely than other methods in high-risk patients. However, while independent of mean BP, its value relies on accurate measurement [19]. Rothwell et al. found a close relationship between long-term BPV and the risk of cardiovascular events and all-cause mortality, even in a subgroup of patients with a systolic BP <140 mmHg [20]. In dialysis patients, visit-to-visit BPV over longer periods of follow-up, which calculated as the coefficient of variation (SD/mean), is widely used to assess long-term BPV, and showed greater prognostic value than average BP or short-term variability in recent studies [20,21]. During our study period, we used this method to evaluate long-term BPV in 175 patients over 12 consecutive months. We found that 48% of these patients showed volume overload (>2 L) over 1-year follow-up, which was severe in nearly 40% of cases, even with strict follow-up and active medical intervention. Although there was no significant difference in mean BP between different volume groups (*p* > 0.05), the long-term systolic and diastolic BPV values were significantly higher in the severe volume overload group than in the other two groups (*p* < 0.05) and correlated positively with the degree of volume overload.

We also sought factors that correlated with BPV in the volume overload group and found a significant positive correlation of BPV with diabetes status, which is consistent with previous reports. Ploumis et al. found that diabetic patients on hemodialysis readily developed fluid retention that was particularly difficult to control. Moreover, research groups led by Ronco and Kwan also found that nearly 22% of diabetic patients on hemodialysis had fluid overload with OH values as high as 4.41 ± 3.72 L, whereas the mean value in their nondiabetic counterparts was only 1.59 ± 1.98 L. The reasons cited for the disorders are as follows: (1) increased fluid intake, stimulated by thirst, and reduced transperitoneal osmotic gradient; (2) ultrafiltration loss secondary to peritoneal membrane hyperpermeability, which may be linked to enhanced inflammation and neoangiogenesis; (3) cardiac insufficiency and atherosclerotic vascular disease, which are common in patients with diabetes independent of age and can cause arterial stiffness and abnormal permeability of blood vessels [22–24].

We also observed that BPV had a close relationship with low adequacy of PD in the volume overload group. Univariate correlation analysis showed that Kt/V and CCr were negatively correlated with BPV while hyperphosphatemia and hyperparathyroidism were positively correlated. Multivariate analysis showed Kt/V and PTH were independently associated with BPV. Decreased adequacy of dialysis can directly lead to fluid overload and contribute to fluctuations in BP. Calcium, phosphate and PTH disturbance is common in dialysis patients. The reasons for the higher serum phosphorus and PTH in patients with severely volume-overloaded are complex, which may be related to factors such as insufficient dialysis due to noncompliance, poor dietary control, and higher blood pressure variation. Excessive serum calcium can cause vascular calcification and deposit in the heart muscle, resulting in cardiac hypertrophy, myocardial interstitial fibrosis and insufficiency. Meanwhile, PTH has a direct repressive effect on cardiac muscle. All these disorders result in structural and functional abnormalities of the cardiovascular system and BP regulation mechanisms [18,25,26].

We also found that BPV in the volume overload group was associated with the severity of protein-energy malnutrition, which exhibited a positive relationship with the normalized protein catabolic rate (nPCR) and an inverse relationship with the SGA score. There were no significant between-group differences in serum albumin, C-reactive protein, or lipid levels. Multivariate analysis showed SGA score was independently associated with BPV. The SGA is a widely used tool to assess nutritional status in peritoneal dialysis patients, which includes subjective assessments about patient’s history of weight loss, presence of anorexia, and vomiting, as well as the physician’s grading of muscle wasting, presence of edema, and loss of subcutaneous fat. These findings are in line with previous reports. Fan et al. studied volume overload in a cohort of nearly 600 patients on PD and found that the degree of volume overload was negatively related with nutritional status and was an independent predictor of quality of life [27]. Persistent fluid overload also causes gastrointestinal mucosal edema and anorexia, both of which inhibit absorption of nutrients, increase the rate of protein catabolism, and accelerate muscle wasting, leading to further fluid and sodium retention. Worsening edema further increases the load on the heart and lungs and leads to sensorimotor impairment. These disorders could have a profound effect on sleep quality and impose a psychological burden on patients, leading to a vicious cycle of impaired BP control [27,28].

The limitations of our study need to be mentioned. First, due to the retrospective and observational design, the conclusions obtained from this study need further confirmation by prospective studies; Second, BCM was measured only one time for each patient at the end of 1-year follow-up period, multiple and regular measurements of BCM can better reflect the patient’s volume over time; Third, the number of participants included in our study was small, thus, our findings need to be confirmed in a larger study population; Fourth, we should pay more attention to improve patient compliance on diet and blood pressure control, even in patients with higher transport qualities, to ameliorate disorders of calcium and phosphate metabolism in PD patients [29–32].

In conclusion, fluid overload is a frequent problem in patients on PD, and reliance on clinical parameters alone for assessment of fluid status might be misleading. The findings of this study indicate that BPV has value as a sensitive and convenient indicator of fluid status in patients on CAPD. Larger studies that include stricter outcome measurements and a longer follow-up period are needed in the future.

## Geolocation information

China.

## Data Availability

Derived data supporting the findings of this study are available from the corresponding author [HR] on request.
